# Inhibitory Effects of Brassicaceae Cover Crop on *Ambrosia artemisiifolia* Germination and Early Growth

**DOI:** 10.3390/plants10040794

**Published:** 2021-04-17

**Authors:** Maja Šćepanović, Marija Sarić-Krsmanović, Valentina Šoštarčić, Ema Brijačak, Josip Lakić, Bojana Špirović Trifunović, Jelena Gajić Umiljendić, Ljiljana Radivojević

**Affiliations:** 1 Faculty of Agriculture, University of Zagreb, Svetosimunska cesta 25, 10000 Zagreb, Croatia; mscepanovic@agr.hr (M.Š.); ebrijacak@agr.hr (E.B.); jlakic@agr.hr (J.L.); 2 Institute for Pesticide and Environmental Protection, Banatska 31 b, 11080 Belgrade, Serbia; marijasaric.msaric@gmail.com (M.S.-K.); pecikos@gmail.com (J.G.U.); ljiljana.radivojevic@pesting.org.rs (L.R.); 3 Faculty of Agriculture, University of Belgrade, 6 Nemanjina Street, 11000 Belgrade, Serbia; spirovic@agrif.bg.ac.rs

**Keywords:** common ragweed, allelopathic potential, *Camelina sativa*, *Raphanus sativus*, *Sinapis alba*, integrated weed control, aqueous extracts, phenolic compounds

## Abstract

Several cover crops (CCs) exert allelopathic effects that suppress weed growth. The aim of the present study was to evaluate the effects of aqueous extracts containing different concentrations [0, 0.5, 1, 2.5, 5, 7.5 and 10% (*w*/*v*)] of Brassicaceae CCs (*Sinapis alba, Raphanus sativus, Camellina sativa)* and of the CCs *Fagopyrum esculentum* and *Guizotia abyssinica* on germination and early growth of *Ambrosia artemisiifolia* L. The allelopathic effects were species and concentration-dependent. *C. sativa*, for example, caused the greatest potential to inhibit germination, shoot, radicle length and fresh seedling weight, whereas *S. alba* and *R. sativus* inhibited germination and early growth of *A. artemisiifolia* only at concentrations ≥7.5%. In contrast, no inhibition was observed when aqueous extracts of *F. escultneum* and *G. abyssinica* were added at any of tested concentration. Liquid chromatography-tandem mass spectrometry detected 15 phenolic compounds in Brassicaceae CCs with the highest content (µg/g) of vanillin (48.8), chlorogenic acid (1057), vanilic acid (79), caffeic acid (102.5) and syringic acid (27.3) in *C. sativa*. Our results suggest that *C. sativa* is the most allelopathic CCs and that the fruits of *C. sativa* are the plant organs richest in allelochemicals.

## 1. Introduction

*Ambrosia artemisiifolia* L. is an invasive alien species that originated in North America and is considered one of the most harmful plant species in Europe [1]. This monoecious species produces up to eight million allergen pollen grains [2]. Although it can grow in a wide range of habitat types and climates [1], the highest annual pollen amounts are measured in Pannonian Plain, i.e., European countries with a continental climate and mid-latitude [3]. In fact, Croatia is among the top three European countries in terms of density of pollen and seeds per area produced by uncontrolled *A. artemisiifolia* plants [4]. In the continental part of Croatia, this species is the most abundant weed species in nearly all summer crops [5] and is usually controlled with herbicides [6]. However, the emergence of herbicide-resistant biotypes worldwide [7], the decreasing number of herbicides available on the market [8], and the generally negative ecotoxicological properties of pesticides, combined with social demands towards pesticide-free agriculture, require other approaches to eliminate weeds from the cultivated landscape. Specifically, *A. artemisiifolia* has developed resistance to several herbicide groups [7] and recently, the poorer efficacy of some postemergence herbicides in soybean has also been reported by Croatian farmers. 

The use of cover crops (CCs) is a well-accepted practice in conversation agricultural systems or organic farming to reduce erosion, increase soil biological activity and prevent nutrient leaching [9,10,11,12,13]. It is also a useful tool for suppressing weed growth [14,15] in those systems since the presence of cover crops is often negatively correlated to weed biomass [9]. Mostly, the effect of cover crops on weed establishment and development can be considered in two ways: their competitive ability and their allelopathic potential by releasing various allelochemicals [10,15,16,17]. The idea of competing vegetation as a sustainable control method for *A. artemisiifolia* has already been demonstrated [18] and further elaborated by a comprehensive field study at 39 sites in Europe, where positive effects of neighboring vegetation in reducing this weed species were demonstrated [4]. However, the possibility of CCs to suppress *A. artemisiifolia* through allelopathy is still unclear. 

Several crops, especially from the Poaceae, Asteraceae and Brassicaceae families, demonstrate strong weed suppression ability by exuding allelochemical compounds from the living plant parts or decomposing residues [9]. Brassicaceae family plants from the *Brassica*, *Camelina*, *Raphanus* and *Sinapis* tribes are frequently cited as allelopathic [19,20]. A promising way to use allelopathy in weed control is using water extracts of allelopathic plants as herbicides [21]. Indeed, high-weed germination inhibition caused by the residues of those species has already been proved in a greenhouse experiment [22] and usually correlated with the presence of different phenols, the most active allelochemicals, in plant tissue [10,20,22,23]. Most allelochemicals, including phenols, are water-soluble, so aqueous extraction is the easiest way to isolate them [24]. Therefore, the aim of the present study is to examine whether aqueous extracts from CCs can indeed inhibit germination and early growth of *A. artemisiifolia*. In addition, to find out the optimal time for the incorporation of CCs, we used one of the most prominent CCs in the study and evaluated which of the plant organs had the highest allelopathic potential. To address this question, the weed was exposed to different concentrations of aqueous extracts of CCs, and the effects on *A. artemisiifolia* germination, germination rate and early growth were examined. To determine whether Brassicaceae CCs in combination with plant species from other families have a higher allelopathic effect as previously suggested [10,14], aqueous extracts of Brassicaceae CCs and CCs from other families were used in the experiment. Since there is no information in the literature on the sensitivity of *A. artemisiifolia* to aqueous extracts of different CCs, several concentrations of each CC were used [20,25] to determine the CC with the highest allelopathic potential. Finally, liquid chromatography-tandem mass spectrometry was used to detect phenolic compounds in CCs and correlate them with their inhibitory potential against *A. artemisiifoilia*.

## 2. Results

### 2.1. Allelopathic Effects of Aqueous Extracts on Germination and Early Growth of A. artemisiifolia 

The suppression of *A. artemisiifolia* germination depended on the type of CC used and the concentration of CC in the aqueous extract. Germination was inhibited only by aqueous extracts of Brassicaceae CCs, *C. sativa* or *S. alba*, or when all CCs were mixed together ((CCMIX), Table 1). At all concentrations ≥2.5%, the aqueous extracts of *C. sativa* significantly inhibited germination to extents ranging from 28.3% to 42.5%. The extracts of *S. alba* and CCMIX at a concentration of at least 10% significantly inhibited germination by 25.6% and 39.1%, respectively.

Similarly, *A. artemisiifolia* shoot length, radicle length and seedling fresh weight were reduced to the greatest extent by 10% extracts of the Brassicaceae *C. sativa*, *R. sativus* and *S. alba* or of all CCs mixed together (Table 2). The data indicate that *C. sativa* aqueous extracts exerted stronger allelopathic effects than the other extracts at nearly all concentrations. For example, at all concentrations (0.5%–10%), the aqueous extract of *C. sativa* resulted in significantly shorter shoots than under control conditions, and the shortening varied from 18.9% to 54.2% with increasing concentration. Similarly, significantly shorter radicles (41.9%–81.8% reduction) and smaller seedlings (23.9%–51.9% reduction) of *A. artemisiifolia* were observed under *C. sativa* extracts but at a concentration ≤ 5%. When all CCs were mixed together (CCMIX), only the radicle of *A. artemisiifiolia* was reduced at concentration ≥5%, but other parameters (shoot and seedling fresh weight) were not affected by any concentration. Moreover, only the highest concentration (10%) of *S. alba* and *R. sativa* extract was required to inhibit all three measured early growth parameters of *A. artemisiifolia*. Finally, the concentrations of *F. esculentum* or *G. abssynica* did not reduce any of the early growth parameters of *A. artemisiifolia*.

### 2.2. Allelopathic Effects of Aqueous Extracts on A. artemisiifolia Germination Rate 

**Figure A1:**
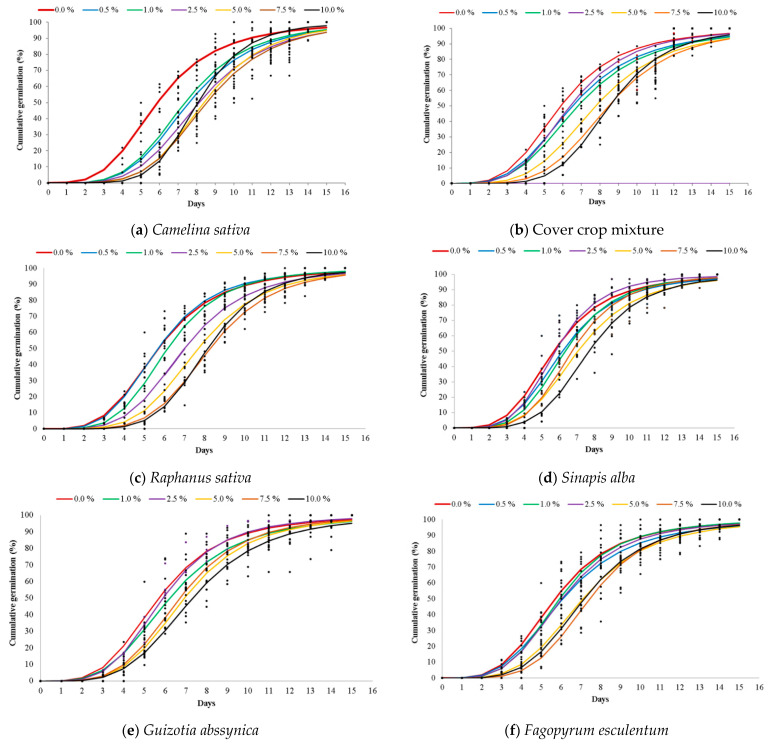
The germination rate of *A. artemisiifolia* upon the aqueous extracts of CCs. The cumulative germination was analyzed using a logistic function in the Bioassay97 statistical program (Onofri 2001), obtaining germination time course under different concentrations of aqueous extracts. The solid line represents the fitted model, and the dots represent observed germination. Within each CC aqueous extract, the same line color the concentrations of aqueous extract (0%–10%).

The extracts differentially affected the germination rate of *A. artemisiifolia* depending on the CCs and concentration. The effect of all CC aqueous extracts was significant for concentrations of 5%. A significant interaction extract concentration x CCs was observed for initial germination (t10) and mean germination (t50) of *A. artemisiifolia*, so the values for each CCs and concentration are shown in Table 3. At concentrations of 0.5%–2.5%, only *C. sativa* delayed the initial germination *(*Figure A1a), while *C. sativa* and CCMIX at these concentrations delayed mean germination, i.e., the number of days at which 50% of the seed sown has germinated (Figure A1a,b). At concentrations of 1% or 2.5%, extracts and almost all Brassicaceae CC delayed the initial or mean germination (Figure A1a,c,d). Additionally, the non-Brassicaceae CCs *F. esculentum* and *G. abssynica* delayed the initial and mean germination at concentrations ≥5% (Figure A1e,f); at concentrations of 0.5% and 1%, *G. abssynica* significantly delayed the mean germination. Taken together, these results indicate that all CCs inhibited germination rate, and *C. sativa* again exerted the strongest effects. 

### 2.3. Allelopathic Effects of Aqueous Extracts of Individual Parts of C. sativa on Germination and Early Growth

Based on the assays of germination and germination rate, *C. sativa* was selected for an assay in which individual plant parts were assessed for their allelopathic effects on *A. artemisiifolia*. The tetrazolium test showed no differences in the percentage of viable seeds between any of the *C. sativa* plant organ aqueous extracts and the control. However, the aqueous extracts of all plant parts (root, stem, leaf, fruit and whole plant) significantly reduced germination, shoot length, radicle length, and seedling fresh weight in almost all cases compared to the control (Table 4). However, the strongest effects against *A. artemisiifolia* were observed with extracts of *C. sativa* fruits, which inhibited germination, shoot length, radicle length and seedling fresh weight by 75%, 57.3%, 90.9% and 75.7%, respectively. These extracts showed an even stronger inhibitory effect on *A. artemisiifolia* than the extracts prepared from all parts of the *C. sativa* plant, which was particularly effective for final germination (75% inhibition when treated with fruit extracts vs. 54% when treated with the extract of the whole plant.) Leaf and root aqueous extracts of *C. sativa* were similar in their effect on early growth inhibition of *A. artemisiifolia*, whereas the stem extract of *C. sativa* only significantly reduced the radical length and seedling fresh weight of *A. artemisiifolia* by respectively, 32.4% and 65.6%.

### 2.4. Chemical Analyses of Aqueous CC Extracts and Correlation with Allelopathic Effects on A. artemisiifolia Radicle Length

Extracts were found to contain 15 phenolic compounds falling into 6 chemical classes (Table 5): phenolic aldehydes, bioflavonoids, flavonoid glycosides, flavonols, hydroxybenzoic acids and hydroxycinnamic acids. Of the total of 15 phenolics detected, 13 were present in the *C. sativa*, *S. alba* and *G. abssynica* extracts; 12 in the *F. esculentum* and *R. sativus* extracts; and 11 in CCMIX. The CC extracts varied significantly in phenolic content, with the following CCs showing significantly more of the indicated phenolics than other CCs: *S. alba*, kaempferol (46.5 µg/g), gallic acid (65.5 µg/g), and p-hydroxybenzoic acid (222.3 µg/g); *F. esculentum*, rutin (1844.3 µg/g), quercetin 8.96 × 10^5^ (895.6 × 10^3^ µg/g), quercetin (135.8 µg/g) and protocatechuic acid (386.3 µg/g); *R. sativa*, p-coumaric acid (84.5 µg/g); *C. sativa*, vanilin (44.8 µg/g), syringic acid (27.3 µg/g) and chlorogenic acid (79.3 µg/g); and CCMIX, caffeic acid (134.8 µg/g), sinapinic acid (11.8 µg/g) and ferulic acid (1143 µg/g).

The most abundant phenol in CC extracts was quercetin, whose concentration ranged from 6.8 × 10^4^–8.95 × 10^5^. The content of most of the other detected phenols was less than 100 µg/g. Sinapinic acid was detected in only two extracts: *S. alba* (4.0 µg/g) and CCMIX (11.8). 

## 3. Discussion

The present study confirmed Brassicaceae CCs as allelopathic plants [19,20] for suppressing the germination and early growth of *A. artemisiifolia. C. sativa*, *S. alba* and *R. sativus* inhibited early growth of *A. artemisiifolia* better than non-Brassicaceae CCs, which is also consistent with previous studies using other weeds as test species [10,22]. However, our study showed that the allelopathic potential of CCs strongly depends on their concentration in the aqueous extracts. With the exception of *C. sativa*, almost all CCs, and especially *F. esculentum* and *G. abssynica*, tested at lower concentrations slightly stimulated the early growth of *A. artemisiifolia*, which has also been explained previously by the fact that the same compound may be inhibitory at a high concentration, stimulatory at a low concentration or have no effect at other concentrations [23]. Among the Brassicaceae CCs tested, we found that *C. sativa* had the strongest inhibitory potential against the germination and early growth of *A. artemisiifolia*. Indeed, its aqueous extracts reduced *A. artemisiifolia* fresh seedling weight by up to 82%, more than any of the other CCs in this study. 

*C. sativa* has usually been analyzed by virtue of its abundant oil content [26] or its use in seed mixtures for wildflower strips [27]. In our study, however, we discovered that it inhibited *A. artemisiifolia* germination and early growth more than any other CCs. As far as we know, this is the first report of *C. sativa* allelopathic effects against *A. artemisiifolia* and the most detailed comparison yet of its allelopathic potential with that of other Brassicaceae CCs. In previous work, aqueous extracts of *C. sativa* roots and shoots inhibited *Avena fatua* germination more strongly than *Brassica napus* did [28]. Another study showed that *C. sativa* strongly inhibited the grass weed species *Echinochloa crus-galli* L. and *Setaria viridis* L., and these effects were associated with phenols in *C. sativa’s* leaves and reproductive organs [22]. The present study also showed the greatest reduction in germination, shoot and root length and seedling fresh weight when *A. artemisiifolia* seeds were treated with aqueous extracts of *C. sativa* fruits, suggesting that flowers are the plant organs richest in allelochemicals. This may suggest that if *C. sativa* is to be incorporated as a cover crop, the best time to do so is the flowering stage [22]. 

The allelopathic activity of Brassicaceae CCs is mediated by glucosinolates as well as by phenolic compounds in plant tissues; these phenolics suppress the synthesis of proteins and nucleic acids, and they inactivate several enzymes in growing plants [29,30,31]. Liquid chromatography-tandem mass spectrometry in the present study indicated the presence of 15 phenols that are candidate mediators of the allelopathy against *A. artemisiifolia* (Table 3). The results of the current study do not allow us to specifically conclude which phenolics most caused growth inhibition of *A. artemisiifolia*, but the phenolics detected here are well-known weed inhibitory agents. For example, vanilic acid damaged dry mass or chlorophyll *a* content of *Echinochloa crus-galli* or *Galinsoga parviflora* to a greater extent than other phenolics did [32]. Syringic acid, for its part, has been found to significantly reduce the seedling growth of *Corchorus olitorius* [33]. Caffeic, syringic and p-coumaric acids appear to help mediate the ability of *B. nigra* to inhibit various weed species [34], and our data suggest that the levels of these compounds in *C. sativa* are more than twice their levels in *B. nigra*. Kaempherol and gallic acid, which were most abundant in our *S. alba* extracts, have also shown allelopathic effects [32,35,36]. Thus, we attribute the sensitivity of early *A. artemisiifolia* growth to *S. alba* aqueous extracts to the presence of kaempherol and gallic acid and the sensitivity to *R. sativus* extracts to an abundance of p-coumaric acid. 

High concentrations of aqueous CC extracts have been shown to cause embryo death in seeds [37], but we did not observe this. None of the tested Brassicaceae CCs completely inhibited *A. artemisiifolia* germination or seedling development. In fact, aqueous *B. nigra* extract at 0.04 g mL^−1^ completely inhibited the germination of *T. alexandrinum*, *P. paradoxa* and *S.officinale* seeds [34], whereas we did not observe that result when we used concentrations even 2.5-fold higher. Indeed, the tetrazolium test did not show differences in the percentage of viable seeds between untreated seeds and seeds treated with extracts from *C. sativa* root, stem leaf, fruit as well as from the whole plant (Table 4). *A. artemisiifolia* may be less sensitive to aqueous CC extracts, which is consistent with the idea that weed species may vary in their sensitivity to allelochemicals [25,38]. Differences in seed size might be a reason, with smaller seeds more susceptible to allelopathic compounds [39]. *A. artemisiifolia* has approximately 10–15-fold greater mass weight (ca. 4–5 g) than many weed species [40]. Further studies should examine whether any CCs can inhibit *A. artemisiifolia* more than the CCs tested here and whether the susceptibility to allelochemicals depends on seed weight, size, texture, color, degree of dormancy or shape [41]. It seems unlikely, however, that allelochemicals should totally inhibit weed growth [12]. 

CCs are usually more allelopathic to weed species when they are applied as a mixture than when they are used on their own [10,14,34,42]. However, this was not the case in the present study. Although CCMIX showed significantly higher content of caffeic, sinapininic and ferulic acids than any of the CCs on their own (Table 3), it did not exert the strongest allelopathic effects on *A. artemisiifolia*. Instead, *C. sativa* exerted stronger effects than CCMIX for all parameters measured (Table 2). Our laboratory results may not be entirely generalizable to the field since field studies suggest that CC mixtures consistently show greater stress tolerance than individual CCs [8]. Nevertheless, our results lead us to suggest increasing the seed proportion of *C. sativa*, the most allelopathic Brassicacea CC, beyond the 5% used in the Terra Gold CC seed mixture. It may even be quite effective to use *C. sativa* alone in fields highly infested with *A. artemisiifolia*, as in Croatia. Indeed, *C. sativa* requires less water than many CCs and resists diseases and pests better [43], making it attractive in crop rotation.

Consistent with its excellent allelopathic potential, *C. sativa* delayed the seed germination of *A. artemisiifolia* even at the lowest concentration of 0.5% (Figure A1a). Delay in seed germination is crucial in weed control and can affect the ability of the seedlings to establish themselves in natural conditions [44]. Thus, under field conditions, *C. sativa* may emerge before *A. artemisiifolia* and develop a competitive advantage over it. This may help explain why the non-Brassicacee CCs *F. esculentum* and *G. abssynica* delayed germination of *A. artemisiifolia* (Figure A1e,f), which, therefore, are also attractive as CCs in the field, even if they showed relatively weak allelopathic effects in our laboratory experiments. 

## 4. Materials and Methods

### 4.1. Plant Material (Sources) 

The commercial CC product Tera Gold 14 (Terra Gold TG-14, Feldsaaten Freudenberger, Krefeld, Germany) was used. It is composed of 40% *Sinapis alba*, 30% *Raphanus sativus* var. *oleiformis*, 20% *Fagopyrum esculentum*, 5% *Camellina sativa* and 5% *Guizotia abyssinica*. The mixture was sown at 25 kg ha^−1^ to a depth of 2–3 cm after wheat harvest at the Šašinovec Experimental Station (45°51′05.2″ N 16°10′34.1″ E) at the University of Zagreb, Faculty of Agriculture, in August 2018. Soil type was silty clay loam (USDA, 1987), with pH 7.74 (H_2_O) and 7.04 (KCl). Aerial parts of the CC mix were harvested separately in October 2018 at the growth stage BBCH 34–87. All CC plants from a 0.5 m × 0.5 m square were collected at five locations. Collected materials were dried at 70 °C in an oven (UF 260, Memmert, Germany) until a constant weight was reached. Dry plant materials were chopped in a blender (GM 300 Grindomix Knife Mill, Retsch), ground in the blender and passed through a 0.5 mm sieve (SM 200, Retsch). 

Mature, ripe seeds (achenes) of *A. artemisiifolia* L. were hand-collected from single populations of plants at the Šašinovec Experimental Station in October 2016. After collection, seeds were cleaned and stored in paper bags in darkness at room temperature until the start of the experiment. Seeds of *A. artemisiifolia* of uniform size and color, without visible signs of insect predation, were selected under a stereomicroscope. *A. artemisiifolia* germination was tested and found to be satisfactory for further seed bioassays.

### 4.2. Seed Bioassay 

The ground plant material was soaked in the dark at room temperature (20 ± 1 °C) and kept in distilled water for 24 h in a 1:10 ratio (0.1 g mL^−1^). The extracts were filtered through two-layer filter paper (80 g/m^2^, 21/N, Munktell) to remove plant debris. The supernatant was stored in the dark at 4 °C until use. Pilot germination tests with aqueous extracts (0.1 g mL^−1^) of CCs, individually or all mixed together, inhibited *A. artemisiifolia* germination, root or shoot development (Table A1). 

Seed bioassays were performed by mixing different amounts of each chosen plant powder in distilled water to concentrations (*w*/*v*) of 0.5%, 1%, 2.5%, 5%, 7.5% or 10%. A plant whose extracts showed the greatest inhibitory effect were further assessed by examining the effects of the aqueous extracts of root, stem, leaf or flower separately. In this plant part bioassay, the tetrazolium test [45] was performed to determine the percentage of viable seeds, i.e., to separate dead from dormant seeds. 

Control Petri dishes contained distilled water. *A. artemisiifolia* seeds were soaked in a 2% KNO_3_ solution for 24 h to alleviate their dormancy. A total of 40 soaked imbibed seeds were placed into each Petri dish (90 mm diameter), to which was added 5 mL of each plant seed solution. Dishes were kept in the climate chamber (HCP 108, Memmert, Germany) under the following conditions: photoperiod, 12 h/12 h; day temperature, 25 °C; night temperature, 15 °C; humidity, 70%; and light intensity, 40–50 μmol/m^2^. All dishes were hermetically sealed with parafilm to avoid evaporation. Germinated seeds were counted every day over a two-week period. On the last day, the percentage of germination and early seedling growth were measured; the latter was measured in terms of shoot length, radicle length and seedling fresh weight. Germination rates were assessed by counting the number of germinated seeds at 24 h intervals during 14 days. Seeds were classified as germinated if they developed radicles > 1 mm. The experimental design was a complete randomized block with four replications.

### 4.3. Chemical Analyses of Aqueous Extracts

#### 4.3.1. Standards and Reagents

Reference standards of phenolic compounds were obtained from Sigma–Aldrich (Steinheim, Germany), Fluka Chemie (Buchs, Switzerland) or Chromadex (Santa Ana, CA, USA). HPLC-grade methanol was purchased from J.T. Baker (Deventer, The Netherlands), and p.a. formic acid and DMSO were obtained from Merck (Darmstadt, Germany).

#### 4.3.2. Liquid Chromatography-Tandem Mass Spectrometry

Water extracts were filtered through 0.45-μm filters of nylon with a diameter of 25 mm (KX Syringe Filter, catalogue no.ESF-NY-25-045-D, Kinesis, www.kinesis-group.com (accessed on 17 April 2021)). Fifteen working standards ranging from 0.001 to 10 μg/mL were prepared by serial 1:1 dilution of standard mixture with a 1:1 mixture of mobile phase A (0.1% aqueous formic acid) and B (0.1% formic acid in methanol). Samples and standards were analyzed using a 1260 Series high-performance liquid chromatography system (Agilent Technologies, Santa Clara, USA) coupled with a 6460A Triple Quad tandem mass spectrometer with an electrospray ion source (Agilent Technologies). This coupled system was controlled using MassHunter software (version B.06.00, Agilent Technologies). Samples (5 μL) were injected into the system, and compounds were separated on a Zorbax Eclipse XDB-C18 column (50 × 4.6 mm, 1.8 μm) at 40 °C. Mobile phase flow rate was 0.4 mL/min, and the following gradient was applied: at 0 min, 5% B; 2 min, 5% B; 10 min, 50% B; 12 min, 70% B; 20 min, 90% B; and re-equilibration time, 5 min. Eluted components were detected using mass spectrometry with the following parameters: nebulization gas (N_2_) pressure, 40 psi; drying gas (N_2_) flow, 11 L/min; temperature, 350 °C; and ion polarity, negative (ESI-). Data for compound detection, used in the dynamic MRM mode (retention time, precursor ion, product ion and Fragment and Collision energies), are given in Table 2. The ion polarity of all compounds was negative. Concentrations of all compounds were determined using the Agilent MassHunter Workstation Software—Quantitative Analysis (ver. B.06.00). 

### 4.4. Statistical Analysis 

The chromatography-mass spectrometry experiments were performed in triplicate. Data were expressed as means ± standard deviation. Cumulative germination on a daily basis was analyzed using a logistic function in the Bioassay97 statistical program (Onofri 2001), and the resulting germination time course was used to determine the time necessary for the germination of 10% (t_10_) or 50% (t_50_) of sown seeds. The normality and homogeneity of variance of all data were checked using the Kolmogorov–Smirnov and Levene tests. Differences in germination percentage, seedling growth, seedling fresh weight and cumulative germination between different CC species and concentrations were assessed for significance using two-way ANOVA using StatSoft, Inc. (2007) STATISTICA data analysis software system, www.statsoft.com (accessed on 17 April 2021), Tulsa OK, USA. Differences between treatments were also assessed for significance using Fisher’s least significant difference test. For all statistical tests, differences associated with *p* < 0.05 were considered significant.

## 5. Conclusions

*A. artemisiifolia* is a highly allergenic species around the world and the most abundant weed species among all summer crops in Croatia. Thus, the present study may help guide future work to examine how the allelopathic effects of CCs vary with abiotic and biotic factors that can degrade allelochemicals in the field. When CCs (*C. sativa*) are incorporated into the soil to provide organic matter and nutrients, our work suggests, at the flowering stage (Table 4). Such work may also guide the effective use of *C. sativa* applied directly as bioherbicides, but further studies are needed to specify which type of phenolic compounds is responsible for growth inhibition of *A. artemisiifolia*. This would be particularly helpful in areas with *A. artemisiifolia*-resistant biotypes [7]. Of course, CCs should be tested for unwanted allelopathy against arable crops [46]. Our preliminary results suggest that aqueous extracts of *C. sativa* do not affect germination or early growth of maize and soybean (data not shown). Consistently, previous work has shown that phenolic acids, even when applied directly at levels much higher than in soil, do not affect seedling growth or early plant growth of maize [47].

## Figures and Tables

**Table 1:** Allelopathic effect of different concentrations of aqueous extracts of cover crops on *A. artemisiifolia* germination.

Extract Concentration (*w*/*v*)	Cover Crop (s)					
	All Crops Mixed (CCMIX)	*C. sativa*	*F. esculentum*	*G. abyssinica*	*R. sativus*	*S. alba*
**Control**	75.00 ± 2.35 ^A–F ab^					
**0.5**	68.13 ± 2.91 ^C^^–F ab^	67.50 ± 2.08 ^C^^–F ab^	76.88 ± 2.81 ^A^^–C a^	79.38 ± 1.25 ^A^^–C a^	77.50 ± 2.54 ^A–C ab^	83.33 ± 3.12 ^A a^
**1.0**	76.88 ± 2.75 ^D–G a^	56.88 ± 1.66 ^E–H bc^	78.13 ± 2.27 ^A–C a^	76.25 ± 2.99 ^A–C a^	78.13 ± 2.73 ^A–C a^	81.67 ± 2.24 ^AB a^
**2.5**	63.75 ± 1.92 ^D–G ab^	53.75 ± 1.51 ^G–I bc^	76.88 ± 2.54 ^A–C a^	74.38 ± 2.39 ^A–D a^	72.50 ± 2.40 ^A–D ab^	72.50 ± 2.40 ^A–D ab^
**5.0**	55.00 ± 1.42 ^G–I bc^	51.25 ± 1.44 ^HI c^	73.75 ± 2.10 ^A–D ab^	72.50 ± 2.40 ^A–D a^	71.25 ± 1.44 ^A–D ab^	69.17 ± 1.61 ^C–E b^
**7.5**	55.00 ± 1.54 ^G–I ab^	48.75 ± 1.07 ^HI c^	69.38 ± 2.15 ^B–D ab^	71.88 ± 2.68 ^A–D a^	68.13 ± 1.53 ^C–F ab^	67.50 ± 1.61 ^C–F bc^
**10.0**	45.63 ± 1.11 ^HI c^	43.13 ± 1.04 ^I c^	63.75 ± 1.44 ^D–G b^	71.88 ± 2.27 ^A–D a^	63.75 ± 1.33 ^D–G b^	55.83 ± 1.24 ^F–H c^

Data are reported as mean ± standard deviation. Differences between different CCs or between different concentrations of the same CC were assessed for significance using two-way analysis of variance. Values with different lowercase letters (a–c) within a column or different uppercase letters (A–I) within a row differ significantly based on Fisher’s least significant difference test at *p* < 0.05. Liquid chromatography-tandem mass spectrometry detected 15 phenolic compounds from 6 different phenolic classes: phenolic aldehydes, bioflavonoids, flavonoid glycosides, flavonols, hydroxybenzoic acid and hydroxycinnamic acid. The phenolic composition and the content of these phenolic compounds (µg/g dry extract) at each CCs are given in Table 5.

**Table 2:** Allelopathic effect of different concentrations of aqueous extracts of cover crops on *A. artemisiifolia* shoot length, radicle length and seedling fresh weight.

Parameter	Concentration (*w*/*v*)	Cover Crop (s)					
		All Crops Mixed Together (CCMIX)	*C. sativa*	*F. esculentum*	*G. abssynica*	*R. sativus*	*S. alba*
**Shoot length (cm)**	**Control**	**1.90 ± 0.52 ^D–N a–c^**					
	0.5	2.34 ± 0.17 ^AB a^	1.54 ± 0.29 ^C d^	1.92 ± 0.67 ^G–K a^	2.09 ± 0.86 ^E–H a^	2.50 ± 0.77 ^CD a^	1.44 ± 0.35 ^L–O bc^
	1.0	2.26 ± 0.20 ^A a^	1.36 ± 0.24 ^C d^	1.81 ± 0.70 ^G–L a^	2.05 ± 0.62 ^F–I a^	2.36 ± 0.74 ^C–F a^	1.59 ± 0.32 ^K–O b^
	2.5	2.23 ± 0.22 ^B a^	1.39 ± 0.11 ^C d^	1.68 ± 0.54 ^I–M a^	2.07 ± 0.62 ^F–I a^	2.00 ± 0.62 ^F–J b^	1.53 ± 0.35 ^J–N b^
	5.0	2.08 ± 0.22 ^C–E a^	1.10 ± 0.15 ^D–G d^	1.69 ± 0.49 ^H–M a^	1.99 ± 0.68 ^F–J a^	1.88 ± 0.34 ^G–K bc^	1.39 ± 0.27 ^M–P bc^
	7.5	1.99 ± 0.23 ^F–J ab^	0.91 ± 0.06 ^P–S e^	1.60 ± 0.43 ^J–N a^	1.97 ± 0.63 ^F–J a^	1.68 ± 0.32 ^I–M c^	1.15 ± 0.26 ^O–R cd^
	10.0	1.52 ± 0.19 ^M–R c^	0.87 ± 0.22 ^S e^	1.64 ± 0.50 ^J–N a^	1.85 ± 0.48 ^G–K a^	1.27 ± 0.22 ^N–R d^	0.98 ± 0.14 ^R–S d^
**Radicle length (cm)**	**Control**	**3.79 ± 0.42 ^D–K a–c^**					
	0.5	4.09 ± 0.25 ^L–O a^	2.69 ± 0.55 ^N–S c^	4.79 ± 0.73 ^A–D a^	4.93 ± 0.57 ^A–C a^	4.41 ± 0.92 ^AB a^	5.46 ± 1.09 ^A a^
	1.0	4.45 ± 0.46 ^L–O c^	2.76 ± 0.28 ^P–T c^	4.62 ± 0.89 ^B–F a^	4.63 ± 0.61 ^B–F ab^	4.08 ± 0.91 ^A–D a^	4.73 ± 0.50 ^A–E ab^
	2.5	4.01 ± 0.39 ^L–O cd^	2.67 ± 0.26 ^P–T cd^	4.49 ± 0.68 ^C–F a^	4.79 ± 0.62 ^A–D ab^	4.05 ± 0.59 ^A–D a^	4.83 ± 0.72 ^A–D ab^
	5.0	2.48 ± 0.18 ^M–P d^	2.20 ± 0.23 ^ST d^	4.46 ± 0.86 ^D–G ab^	4.32 ± 0.33 ^D–H ab^	3.65 ± 0.37 ^G–J b^	3.86 ± 0.47 ^F–J bc^
	7.5	2.00 ± 0.40 ^M–R d^	1.01 ± 0.61 ^T e^	3.28 ± 0.58 ^I–K bc^	3.94 ± 0.39 ^E–I bc^	2.35 ± 0.17 ^L–N c^	3.61 ± 0.40 ^H–J c^
	10.0	1.32 ± 0.35 ^O–T e^	0.69 ± 0.28 ^T e^	2.27 ± 0.40 ^K–M c^	3.05 ± 0.21 ^J–L c^	1.23 ± 0.29 ^R–T d^	1.59 ± 0.53 ^N–T d^
**Seedling fresh weight (g)**	**Control**	**0.27 ± 0.05 ^A–K ab^**					
	0.5	0.40 ± 0.17 ^C–I a^	0.31 ± 0.04 ^B–G a^	0.30 ± 0.03 ^B–F a^	0.33 ± 0.07 ^A–D a^	0.32 ± 0.04 ^A–E a^	0.34 ± 0.04 ^A–C a^
	1.0	0.49 ± 0.11 ^A a^	0.23 ± 0.07 ^E–I a^	0.30 ± 0.01 ^B–F a^	0.35 ± 0.07 ^A–C a^	0.33 ± 0.04 ^A–E a^	0.36 ± 0.01 ^AB a^
	2.5	0.43 ± 0.13 ^A–C a^	0.22 ± 0.07 ^G–K a^	0.31 ± 0.06 ^A–F a^	0.33 ± 0.09 ^A–E a^	0.29 ± 0.06 ^B–F ab^	0.35 ± 0.04 ^A–C a^
	5.0	0.30 ± 0.16 ^I–K b^	0.20 ± 0.11 ^G–K c^	0.33 ± 0.04 ^A–E a^	0.32 ± 0.04 ^A–F a^	0.29 ± 0.04 ^B–F ab^	0.33 ± 0.06 ^A–D ab^
	7.5	0.27 ± 0.17 ^KLb c^	0.17 ± 0.01 ^M c^	0.32 ± 0.05 ^A–D a^	0.31 ± 0.03 ^A–F a^	0.23 ± 0.01 ^F–J bc^	0.31 ± 0.03 ^A–F ab^
	10.0	0.22 ± 0.15 ^J–L bc^	0.13 ± 0.09 ^LM c^	0.27 ± 0.03 ^B–H a^	0.28 ± 0.03 ^B–G a^	0.18 ± 0.02 ^H–K c^	0.25 ± 0.01 ^D–I c^

Data are reported as mean ± standard deviation. Differences between different CCs or between different concentrations of the same CC were assessed for significance using two-way analysis of variance. Values with different lowercase letters (a–e) within a column or different uppercase letters (A–T) within a row differ significantly based on Fisher’s least significant difference test at *p* < 0.05. Liquid chromatography-tandem mass spectrometry detected 15 phenolic compounds from 6 different phenolic classes: phenolic aldehydes, bioflavonoids, flavonoid glycosides, flavonols, hydroxybenzoic acid and hydroxycinnamic acid. The phenolic composition and the content of these phenolic compounds (µg/g dry extract) at each CCs are given in Table 5.

**Table 3:** The effect of aqueous extracts of CCs on the number of days needed for 10% (t_10_) or 50% (t_50_) of *A. artemisiifolia* seeds to germinate.

Aqueous Extract Concentration (*w*/*v*)	Cover Crop (s)											
	All Crops Mixed Together (CCMIX)		*C. sativa*		*F. esculentum*		*G. abssynica*		*R. sativus*		*S. alba*	
	t_10_	t_50_	t_10_	t_50_	t_10_	t_50_	t_10_	t_50_	t_10_	t_50_	t_10_	t_50_
**Control**	3.156 ^PR ab^	5.520 ^T a^	3.205 ^PR ab^	5.882 ^P–T a–c^	3.245 ^PR ab^	5.717 ^R–T ab^	3.067 ^R a^	5.575 ^ST a^	3.152 ^PR ab^	5.615 ^ST a^	3.237 ^PR ab^	5.605 ^ST a^
0.5	3.487 ^O–R a–c^	6.514 ^I–R c–g^	4.614 ^E– I h–l^	7.597 ^C–G k–p^	3.390 ^O–R a–c^	6.120 ^M–T a–e^	3.595 ^N–R a–d^	8.210 ^K–S o–t^	3.296 ^PR ab^	5.692 ^R–T ab^	3.514 ^O–R a–c^	6.163 ^L–T a–e^
1.0	3.729 ^L–R b–f^	6.828 ^G–N e–j^	4.596 ^E–I h–l^	7.437 ^D–H i–n^	3.515 ^O–R ab^	5.983 ^O–T a–d^	3.634 ^M–R a–e^	6.400 ^K–S b–f^	3.785 ^K–P b–g^	6.165 ^L–T a–e^	4.003 ^I–O c–h^	6.380 ^K–Sb–f^
2.5	3.632 ^M–R a–e^	6.681 ^D–H d–h^	5.036 ^B–E l–o^	8.177 ^A–D n–s^	3.484 ^O–R a–c^	6.058 ^N–T a–d^	3.522 ^O–R a–c^	5.928 ^O–T a–c^	4.410 ^E–K g–l^	6.946 ^F–M f–k^	3.579 ^N –R a–d^	5.774 ^R–Ta–c^
5.0	4.522 ^E–I h–l^	7.787 ^A–E m–s^	5.607 ^AB op^	8.447 ^AB r–t^	4.286 ^G–M e–j^	7.171 ^E–K g–m^	4.223 ^H–N d–i^	6.954 ^F–L f–l^	4.908 ^C–G j–n^	7.695 ^B–F l–p^	4.211 ^H–N d–i^	7.076 ^E–K f–m^
7.5	5.323 ^A–D m–p^	8.473 ^AB st^	5.529 ^A–C n–p^	8.591 ^A t^	4.824 ^D– H i–m^	7.468 ^C–H j–o^	4.037 ^I–O c–h^	6.697 ^H–P d–i^	5.473 ^A–D m–p^	8.293 ^A–C p–t^	4.237 ^H– N d–i^	6.727 ^H–O d–j^
10.0	5.810 ^A p^	8.549 ^A t^	5.895 ^A p^	8.163 ^A–D n–t^	4.445 ^E– K g–l^	7.180 ^E–K g–m^	4.368 ^F–L f–k^	7.316 ^E–I h–m^	5.658 ^AB op^	8.179 ^A–D n–t^	5.024 ^B–F k–o^	7.718 ^B–F m–r^

Data are reported as mean ± standard deviation. Differences between different CCs or between different concentrations of the same CC were assessed for significance using two-way analysis of variance. Values with different lowercase letters (a–t) within a column or different uppercase letters (A–T) within a row differ significantly based on Fisher’s least significant difference test at *p* < 0.05. Liquid chromatography-tandem mass spectrometry detected 15 phenolic compounds from 6 different phenolic classes: phenolic aldehydes, bioflavonoids, flavonoid glycosides, flavonols, hydroxybenzoic acid and hydroxycinnamic acid. The phenolic composition and the content of these phenolic compounds (µg/g dry extract) at each CCs are given in Table 5.

**Table 4:** Effect of aqueous extracts of individual parts of *C. sativa* at 0.1 g mL^−1^ on *A. artemisiifolia* weed radicle length, coleoptile length and whole fresh seedling weight.

Parameters	Control	*Camelina sativa Aqueous Extracts*				
		Root	Stem	Leaf	Fruit	Whole Plant
Viable seed (%)	95.2 ± 5.22 ^a^	95.3 ± 3.01 ^a^	93.3 ± 3.27 ^a^	95.2 ± 6.57 ^a^	92.8 ± 5.22 ^a^	90.7 ± 6.02 ^a^
Germinated seed (%)	73.6 ± 6.69 ^a^	58.7 ± 10.33 ^b^	63.3 ± 5.89 ^a,b^	55.2 ± 7.69 ^b^	18.4 ± 8.76 ^c^	54.0 ± 10.04 ^b^
Shoot length (cm)	1.43 ± 0.11 ^a^	0.65 ± 0.36 ^b^	1.25 ± 0.57 ^a^	0.66 ± 0.39 ^b^	0.61 ± 0.40 ^b^	0.52 ± 0.34 ^b^
Radical length (cm)	3.55 ± 0.70 ^a^	0.95 ± 0.47 ^b,c^	1.22 ± 0.69 ^b^	1.06 ± 0.46 ^b^	0.32 ± 0.13 ^d^	0.41 ± 0.09 ^c,d^
Seedling fresh weight (g)	0.37 ± 0.07 ^a^	0.16 ± 0.07 ^c^	0.25 ± 0.07 ^b^	0.14 ± 0.07 ^c^	0.09 ± 0.04 ^c^	0.12 ± 0.05 ^c^

Data are reported as mean ± standard deviation. The experiment was performed in 4 replicates of 25 seeds each and performed twice. Differences between different plant parts of *C. sativa* on germination and early growht of *A. artemisiifolia* were assessed for significance using one-way analysis of variance (ANOVA) completed with Fisher’s least significant difference (LSD) test, *p* < 0.05. Means in the same row marked by different letters (a–d) differ significantly (*p* < 0.05). Liquid chromatography-tandem mass spectrometry detected 13 phenolic compounds from 5 different phenolic classes in *C. sativa* dry extracts: phenolic aldehydes, flavonoid glycosides, flavonols, hydroxybenzoic acid and hydroxycinnamic acid. The phenolic composition and the content of these phenolic compounds (µg/g dry extract) are given in Table 5.

**Table 5:** Quantification of phenolics in aqueous extracts from six cover crops.

Chemical Class	Compound	Content of Phenolic Compounds (µg/g of Dry Extract)					
		*S. alba*	*F. esculentum*	*R. sativa*	*G. abssynica*	*C. sativa*	CCMIX
**Phenolic aldehydes**	Vanillin	19.0 ± 0.28 ^b^	13.3 ± 0.22 ^c^	19.0 ± 0.23 ^b^	13.8 ± 0.11 ^c^	44.8 ± 0.39 ^a^	n.d. ^d^
**Bioflavonoids**	Kaempferol	46.5 ± 0.59 ^a^	n.d. ^c^	n.d. ^c^	40.0 ± 0.44 ^b^	n.d. ^c^	n.d. ^c^
**Flavonoid glycosides**	Rutin	n.d. ^e^	1844.3 ± 1.48 ^a^	6.0 ± 0.09 ^d^	28.8 ± 0.25 ^c^	413.5 ± 0.86 ^b^	416.5 ± 0.87 ^b^
**Flavonols**	Quercetin	71.6 × 10^3^ ± 21.18 ^d^	895.6 × 10^3^ ± 10.90 ^a^	189.9 × 10^3^ ± 25.15 ^b^	980.5 ± 35.65 ^e^	68.8 ± 0.75 ^f^	88.8 × 10^3^ ± 5.25 ^c^
	Quercitin	40.8 ± 0.86 ^c^	135.8 ± 1.11 ^a^	8.3 ± 0.05 ^e^	7.0 ± 0.09 ^e^	33.3 ± 0.48 ^d^	59.5 ± 0.60 ^b^
**Hydroxybenzoic acids**	Gallic acid	65.5 ± 0.47 ^a^	32.3 ± 0.65 ^b^	18.5 ± 0.43 ^c^	17.5 ± 0.34 ^c^	17.5 ± 0.35 ^c^	26.3 ± 0.50 ^b^
	Protocatechuic acid	55.5 ± 0.60 ^e^	386.3 ± 0.74 ^a^	63.5 ± 0.76 ^d^	38.3 ± 0.53 ^f^	100.5 ± 0.50 ^c^	113.5 ± 0.52 ^b^
	p-Hydroxybenzoic acid	222.3 ± 0.86 ^a^	38.5 ± 0.38 ^c^	154.3 ± 1.0 ^b^	35.8 ± 0.43 ^c^	36.8 ± 0.45 ^c^	27.0 ± 0.43 ^d^
	Syringic acid	7.0 ± 0.15 ^d^	12.3 ± 0.11 ^c^	6.8 ± 0.10 ^d^	13.0 ± 0.10 ^b^	27.3 ± 0.11 ^a^	n.d. ^e^
	p-coumaric acid	25.3 ± 0.53 ^c^	26.5 ± 0.43 ^c^	84.5 ± 0.59 ^a^	16.3 ± 0.24 ^d^	74.8 ± 0.61 ^b^	29.5 ± 0.30 ^c^
**Hydroxycinnamic acids**	Chlorogenic acid	n.d. ^d^	87.8 ± 0.47 ^c^	n.d. ^d^	37.5 ± 0.33 ^c^	1057.0 ± 1.58 ^a^	587.8 ± 1.20 ^b^
	Vanillic acid	31.8 ± 0.56 ^c^	n.d. ^d^	37.0 ± 0.22 ^b^	n.d. ^d^	79.3 ± 0.65 ^a^	n.d. ^d^
	Caffeic acid	14.5 ± 0.11 ^f^	46.3 ± 0.38 ^c^	26.8 ± 0.30 ^e^	35.0 ± 0.52 ^d^	102.5 ± 0.65 ^b^	134.8 ± 0.60 ^a^
	Sinapinic acid	4.0 ± 0.09 ^b^	n.d. ^c^	n.d. ^c^	n.d. ^c^	n.d. ^c^	11.8 ± 0.07 ^a^
	Ferulic acid	39.8 ± 0.34 ^c^	11.8 ± 0.19 ^d^	552.0 ± 1.11 ^b^	2.5 ± 0.05 ^e^	35.5 ± 0.33 ^c^	1143.0 ± 2.86 ^a^

n.d.—not detected (below the limit of quantification of 0.5 µg/mL). Data are reported as mean ± standard deviation. Differences between different CCs were assessed for significance using one-way analysis of variance. Values with different lowercase letters (a–f) within a row differ significantly based on Fisher’s least significant difference test at *p* < 0.05.

## Appendix A

**Table A1:** Optimized dynamic MRM parameters.

Compound	Precursor *m*/*z*	Product *m*/*z*	(Fr) (V)	CE (V)	t_R_ (min)
Gallic acid	169.0	125.0	90	10	3.67
Protocatechuic acid	153.0	109.0	105	9	6.27
Chlorogenic acid	353.0	191.0	100	10	8.54
Vanillic acid	167.0	108.0	100	15	9.14
Caffeic acid	179.0	135.0	100	10	9.14
Syringic acid	197.0	182.0	90	7	9.71
Vanilin	151.1 151.1	136.0 90.0	100 100	17 21	10.02
p-coumaric acid	163.0	119.0	90	9	10.68
Sinapinic acid	223.0	193.0	100	17	11.13
Ferulic acid	193.0	134.0	90	11	11.14
Rutin	609.0	300.0	135	42	11.96
Quercetin	301.0	151.0	130	15	13.68
Quercitin	447.0	300.0	190	27	12.07
Kaempferol	285.0	285.0	130	0	14.25

**Table A2:** The effects of different aqueous extracts on germination, shoot and radical length of *A. artemisiifolia* seeds.

Cover Crop (s)	Parameters		
	Germination (%)	Shoot Length (cm)	Radical Length (cm)
Control	35.0 ± 8.08 ^a^	3.06 ± 0.89 ^a^	2.51 ± 0.42 ^a^
CCmix	23.0 ± 3.06 ^b^	0.90 ± 0.05 ^c^	1.05 ± 0.11 ^c^
*S. alba*	8.0 ± 1.90 ^c^	0.63 ± 0.04 ^c,d^	0.10 ± 0.00 ^d^
*R. sativus*	1.0 ± 0.06 ^d^	0.00 ± 0.00 ^e^	0.27 ± 0.08 ^d^
*F. esculentum*	12.0 ± 1.32 ^c^	2.29 ± 0.09 ^b^	1.98 ± 0.01 ^b^
*C. sativa*	9.0 ± 0.50 ^c^	0.31 ± 0.04 ^d^	0.08 ± 0.03 ^d^
*G. abssynica*	9.0 ± 1.72 ^c^	0.92 ± 0.08 ^c^	0.82 ± 0.04 ^c^

Data are reported as mean ± standard deviation. Differences between different CCs were assessed for significance using one-way analysis of variance. Values with different lowercase letters (a–e) within a column differ significantly based on Fisher’s least significant difference test at *p* < 0.05.
